# Brazilian silverside, *Atherinella brasiliensis* (Quoy & Gaimard,1825) embryos as a test-species for marine fish ecotoxicological tests

**DOI:** 10.7717/peerj.11214

**Published:** 2021-04-14

**Authors:** Natália Martins Feitosa, Emiliano Nicolas Calderon, Rhennã Nascimento da Silva, Sônia Lopes Rezende de Melo, Jackson Souza-Menezes, Rodrigo Nunes-da-Fonseca, Márcia Vieira Reynier

**Affiliations:** 1 Laboratório Integrado de Ciências Morfofuncionais, Instituto de Biodiversidade e Sustentabilidade (NUPEM), Universidade Federal do Rio de Janeiro, Macaé, RJ, Brazil; 2 Laboratório Integrado de Biociências Translacionais, Instituto de Biodiversidade e Sustentabilidade (NUPEM), Universidade Federal do Rio de Janeiro, Macaé, RJ, Brazil; 3 Petrobras Research and Development Center (CENPES), PETROBRAS, Rio de Janeiro, RJ, Brazil; 4 LABTOX—Laboratório de Análise Ambiental Ltda, Rio de Janeiro, RJ, Brazil; 5 Programa Pós-Graduação em Ciências Ambientais e Conservação (PPG-CiAC), Universidade Federal do Rio de Janeiro, Macaé, RJ, Brazil

**Keywords:** *Atherinella brasiliensis*, Brazilian silverside embryos, Developmental toxicity tests, Marine fish embryo test

## Abstract

The fish embryo test (FET) is an alternative to the classic freshwater toxicity test used to assess environmental hazards and risks to fish. This test has been standardized and adopted by the Organization for Economic and Cooperation and Development (OECD). As salinity may affect the substances’ toxicity, we describe the development of an alternative euryhaline test species for embryonic ecotoxicological tests: the Brazilian silverside *Atherinella brasiliensis* (Quoy & Gaimard, 1825). This species is broadly distributed along the coast of South America and is able to inhabit a broad range of environmental and saline conditions. Ours is the first study on the maintenance of a native South American species for natural reproduction and the generation of embryos for tests. The embryos used are transparent and possess fluorescent cells which have only been seen in a few species and which may be used as markers, making it an alternative assessment tool for the lethal and sublethal substances in marine and estuarine environments. We provide a detailed description and analysis of embryonic development under different salinities and temperatures. The embryos and larvae developed in similar ways at different salinities, however as temperatures increased, mortality also increased. We considered the effects of the reference toxicants Zn^2+^ and SDS using a protocol similar to the FET that was standardized for zebrafish. Brazilian silverside embryos are as sensitive as freshwater, or euryhaline fish, to the surfactant but are more resistant to metals prior to hatching. We were able to show the advantages of the Brazilian silverside as a model for a marine fish embryo test (FETm) with high levels of reproducibility and little contaminated waste.

## Introduction

After decades of ecotoxicological studies, our ability to detect the impact of substances in our environment is still insufficient (Werner & Hitzfeld, 2012). The fish embryo test (FET) has been standardized and adopted as an alternative to the acute fish toxicity test for environmental hazard and risk assessment as the OECD TG236 test by the Organization for Economic and Cooperation and Development (OECD) (Braunbeck et al., 2005, 2014; OECD, 2013). The zebrafish (*Danio rerio*) used in FETs is one of the most studied fish models in a wide range of scientific fields around the world. The availability of the zebrafish genome enabled the identification of gene-encoding biomarkers that are responsive to toxic compounds. These biomarkers can also be identified in mammalian vertebrates (Carvan, Incardona & Rise, 2008).

The zebrafish is a freshwater fish and it should be noted that according to a number of studies (Borecka et al., 2016; Hall & Anderson, 1995; Latała, Nędzi & Stepnowski, 2010) salinity may influence the toxicity of substances to freshwater organisms. For example, an increase in salinity may decrease the bioavailability of metals, such as zinc, leading to hazardous effects on euryhaline fish versus freshwater fish (Bielmyer et al., 2012). This example highlights the need for vertebrate species from environments with different salinities to be used in toxicity testing.

Ecotoxicological tests have been performed using embryos and/or larvae of euryhaline and marine species in central North America, Europe, and Asia. Some of these species were established as part of standardized fish testing methods used by environmental agencies, and include the Atlantic silverside (*Menidia menidia*), the Inland silverside (*Menidia beryllina*), and the sheepshead minnow (*Cyprinodon variegatus*) (USEPA, 2002a, 2002b, 2002c). Other species important to ecotoxicological studies include the Pacific herring (*Clupea pallasii*), pink salmon (*Oncorhynchus gorbuscha*) (Dinnel et al., 2011; Incardona et al., 2015), Indian medaka (*Oryzias melastigma*) (Dong et al., 2014), and Atlantic haddock (*Melanogrammus aeglefinus*) (Sørhus et al., 2015, 2017). However, testing methodologies for these species are not standardized for age, sample size or number, water volume, and time as they are for the zebrafish FET (OECD, 2013) and some species cannot be feasibly maintained in an ordinary research facility. As there is no representative species from the local areas in South American tropical waters, it is necessary to establish an alternative fish species for ecotoxicological studies.

Until recently, only acute toxicity test methods for the adults of freshwater species *Danio rerio* and the embryo/larvae of *Pimephales promelas* were standardized by the Brazilian Association of Technical Standards (ABNT, 2016). Martins & Bianchini (2011) called attention to the issue in Brazil that “native species are shown to be in most cases more sensitive to environmental pollutants than foreign or exotic species belonging to the same group. Therefore, there is a need for standardization of protocols and selection of native species for use in toxicity tests”. We established the maintenance of some specimens of the euryhaline fish Brazilian silverside *Atherinella brasiliensis* (Quoy & Gaimard, 1825) in the laboratory in order to study the effects of different substances at different salinities. The fish have an elongated body with a silvery lateral line (França et al., 2007). The features of the Brazilian silverside, such as its broad distribution along the coast at different environments, feeding behavior, and sensitivity to environmental pollution, demonstrate a plasticity to inhabit in a broad range of environmental conditions, making it an ideal species to establish in the laboratory.

The Brazilian silverside lives along most parts of the South American Atlantic coast, from Venezuela to southern Brazil. They are found mostly in brackish water in estuarine and coastal areas and are prey for commercial fish species (Menezes et al., 2003). Studies on species biology along the coast were performed in Rio Grande do Sul (RS), Rio de Janeiro (RJ), and Pernambuco (PE) states, including the environmentally protected Jurubatiba Restinga lagoons located north of RJ (Bervian & Fontoura, 2007; Di Dario et al., 2013; Favaro, Lopes & Spach, 2003; França et al., 2007). Reproductive peaks happen at different times of the year depending on the latitude, which may be related to the temperature. The lower the latitude, the higher the temperature (Fávaro, Oliveira & Verani, 2007). For example, the reproductive peak in Rio de Janeiro happens in a broader season that can be in July (Barbien et al., 1990) or September to December (Cardoso, 2007), while to the south in Rio Grande do Sul, the peak season is in November (Fávaro, Oliveira & Verani, 2007). The early larval stages and adults are opportunistic feeders, where the diet includes plant detritus, algae, zooplankton, insect or fish eggs, etc (Chaves & Vendel, 2008; Contente, Stefanoni & Spach, 2010; Rocha, Silva-Falcão & Severi, 2008). Although the Brazilian silverside can be found in some areas presenting anthropogenic activities, a study conducted at four beaches of Guanabara Bay in RJ showed that they were absent on beaches with the influences of waves, high anthropogenic pressures, or the presence of fishermen and bathers (Franco et al., 2016). *A. brasiliensis* was indicated as an adequate sentinel species for pollution, since it presented decreased capacity of osmoregulation and high levels of cortisol even 7 months after an oil spill at Paranaguá Bay in 2004 (Souza-Bastos & Freire, 2011). These characteristics demonstrate that the Brazilian silverside is an omnivorous species with some plasticity but is also sensitive to environmental changes.

The first reports on the early stages of life of the Brazilian silverside were conducted with gametes artificially obtained by abdominal massage for in vitro fertilization (Del Río et al., 2005) or with larvae collected by ichthyoplankton sampling (França et al., 2007). We obtained the embryos by natural spawning and some mature adult specimens were kept for reproduction. The embryolarval development was analyzed and specimens were raised to adulthood until F2s were generated. Some morphological aspects, like the presence of fluorescent cells that can be used as cell markers, were reported. Our study may help establish a line of an Atlantic South American representative species in any facility overseas. For better reproducibility, static experiments were performed in 24 well plates similarly to the OECD FET protocols. Tests were done at different salinities and temperatures, and generated small volumes of contaminated wastes. The embryo’s sensitivity to the reference substances (sodium dodecyl sulfate and zinc) at a marine salinity of 35ppt compared with other euryhaline and freshwater species demonstrate its potential for risk assessment analyses of substances in marine/estuarine environments. Altogether demonstrate that, the Brazilian silverside *A. brasiliensis* is proposed as a test species for a marine fish embryo testing (FETm) in ecotoxicology.

## Materials and Methods

### Fish collection

Forty adult *Atherinella brasiliensis* fish were captured using a seine net in Rio São João (22°34′S; 041°59′W) in Casimiro de Abreu City, RJ; Carapebus Lagoon (22°14′S; 041°35′W) in Carapebus City, RJ; and Garças Lagoon (22°12′S; 041°29′W) in Quissamã City, RJ. The former two locations are found in the National Park of Jurubatiba Restinga, Rio de Janeiro State, Brazil. We obtained permission for fish collection from the Brazilian Ministry of the Environment (MMA)/Chico Mendes Institute for Biodiversity Conservation (ICMBio) (Permits n° 43874-1 and 43874-2). Careful attention was paid to limit the time the fish were kept out of the water during their removal since more than 2 min out of the water can be fatal. Fish were transported to the laboratory in 50 L thermally-insulated boxes filled with 25 L of local water (10–25 salinity) with an air pump system.

### Fish maintenance and breeding

The fish were gradually acclimated to 20 ± 1 salinity with artificial seawater (red sea salt from Red Sea or Instant Ocean® Sea Salt) or by adding filtered water (with perlon and activated carbon), and the pH naturally ranged between 6.5 and 7.3. Adult fish were maintained in 130 L glass aquariums filled with water at 20 ± 1 salinity passing through a biological filtration system, activated carbon filter, and a Macro aqua hanging skimmer. The water temperature was kept at 24 ± 1 °C in a room with a 14 h light/10 h dark photoperiod. Artificial plants were introduced into the aquariums to allow for egg fixation. Ten to 15 individuals were kept in each aquarium and were fed three times a day with Sera Vipan^®^, Sera Vipagran^®^, and Alcon Basic^®^. Adults longer than 8 cm were given smooth abdominal massages to identify their sex after gamete release and identification. Eggs that are round and transparent are more readily identifiable than sperm, which is white and more viscous. The gametes were released naturally into the water when the lights were off and the eggs were collected by hand from the filter and the artificial plants. The collected eggs were kept in a 10 cm petri dish with aquarium water and were taken to the laboratory to clean the filaments and for test preparation. Fertilized eggs were used in the ecotoxicological tests and for culture maintenance. The larvae obtained in the laboratory were returned to the adult aquarium in 100 mm thick, 15 cm high PVC pipes to be raised to adulthood. The bottom of the pipes was covered with voil tissue of 47 g/m^2^ and were hung on the glass side of the adult aquarium. The larvae were fed with Sera micron daily until they reached 5–10 mm, at which point artemia nauplii was added into their daily diet. The juvenile fish were transferred into their own aquarium when they reached 1.5–2.0 cm, after more or less 2 months, and were then fed the same diet as the adults. Maturity was considered to be achieved when the first eggs were found in the plants and pumps.

### Embryo tests: salinity, temperature, and toxicity

Fertilized eggs obtained from captured fish were added to the experiments until the blastula stage, 9 to 10 h postfertilization (9–10 hpf) at 25 ± 1 °C. Eggs had their filaments removed using microscissors to improve the visualization of the embryos and were washed five times in an embryo medium (EM) to remove excess of dirt. The filaments are adherent and easily collect contaminants that contain microorganisms that will compete for oxygen. The EM was prepared by dissolving artificial sea salt in distilled water and was autoclaved prior to use.

*Atherinella brasiliensis* embryo development was assessed using different salinities (15, 20, 25, 30, and 35 ppt) at 25 and 28 °C.

The salinity differences tested did not affect embryo development. Therefore, reference substance tests were conducted using zinc sulfate heptahydrate (CAS 7446-20-0) and sodium dodecyl sulfate (SDS) (CAS 151-21-3) at 35 ppt salinity to analyze their toxicity in a marine environment. Zinc sulfate stock solutions were prepared in distilled water and then diluted in EM. SDS stock solutions were prepared directly in EM. Test concentrations nominally ranged from 1.0 to 10,000 mg/L^−1^ for zinc and 0.4 to 5.0 mg/L^−1^ for SDS, in a geometric series that included four concentrations and a control group.

Salinity, temperature, and reference substance experiments were static and were conducted under the same conditions, using a 24-well plate containing 2 mL of test solution and one embryo per well. Ten replicates per experimental condition were used, and each experiment was repeated three times. The 24-well plates were sealed with PVC membrane foil to minimize evaporation. The exposure period was 12 days (from fertilized eggs to larvae), and the maximum salinity variation was 2 points until the end of each experiment. Embryos were observed daily under a stereomicroscope for morphological abnormalities and mortality. Our protocol was approved by CEUA n°063/17.

### Image capture

Embryonic/larval images were obtained using the stereo microscope Leica M205FA. A single image was obtained using the combination of pictures taken from a Z stack. The fluorescent cells were analyzed under UV light combining excitation and barrier filters. The green-yellowish fluorescence from the cells was analyzed under a 425/60 nm (395–455 nm) GFP excitation filter and a 480 LP barrier filter, while the red fluorescence was analyzed under a 545/30 nm (510–560 nm) DsRED excitation filter and a 620/60 nm (590–650 nm) barrier filter.

### Statistical analysis

We evaluated the differences in mortality rates and hatching eggs among treatments using one-way and two-way analysis of variance (ANOVA) with salinity, temperature, SDS concentration, and Zn concentration as independent factors, followed by a Tukey HSD post hoc test (Zar, 2010). Normality and homogeneity of variances were tested using Kolmogorov–Smirnov and Levene’s tests, respectively (Zar, 2010). The FET OECD test guidelines outline the lethal endpoints (OECD, 2013) and only coagulation was observed along the tests and was used to calculate LC50. The data were presented as the mean ± SE. The 24 h, 96 h, and 12 dpf lethal concentrations (LC50) were calculated using the PriProbit analysis program (Sakuma, 1998). These LC50 at 24 h, 96 h, and 12 dpf were calculated according to the most sensitive timepoints observed for *A. brasiliensis* (24 h and 12 dpf) and 96 h was compared with other fish tests from the literature (Aalders et al., 2016; Bielmyer et al., 2012; USEPA, 2002b, 2002c).

## Results

### Reproduction

The sex ratio in the aquarium was 1:1, which was similar to previous observations in natural populations (Cardoso, 2007; Fávaro, Oliveira & Verani, 2007). The number of eggs produced by the adults varied from 150 to 1,000 eggs on spawning days, and the embryos were generally synchronized at the same stage. The first eggs were found and collected from the aquarium of lab raised fish when the specimens were 8–9 months of age and 6 to 8 cm in length (Fig. S1). The maturation size for *A. brasiliensis* in this study was consistent with previous findings from environmental studies, indicating that mature adults are eight to nine cm in length at approximately 1 year old (Bervian & Fontoura, 2007; Favaro, Lopes & Spach, 2003).

### Development

The morphological analysis of the embryos along a FETm describes *A. brasiliensis* ontogeny from the one-cell stage until their hatching at 25 ± 1 °C. Filaments from the chorions were removed to highlight the morphological aspects. The terms used here for staging were similar to zebrafish (Kimmel et al., 1995) to facilitate a comparison with FET, although the morphology of the embryo during development was similar to medaka *Oryzias latipes* (Iwamatsu, 2004). The time of development was indicated by hours postfertilization (hpf) and days postfertilization (dpf).

**Figure 1:**
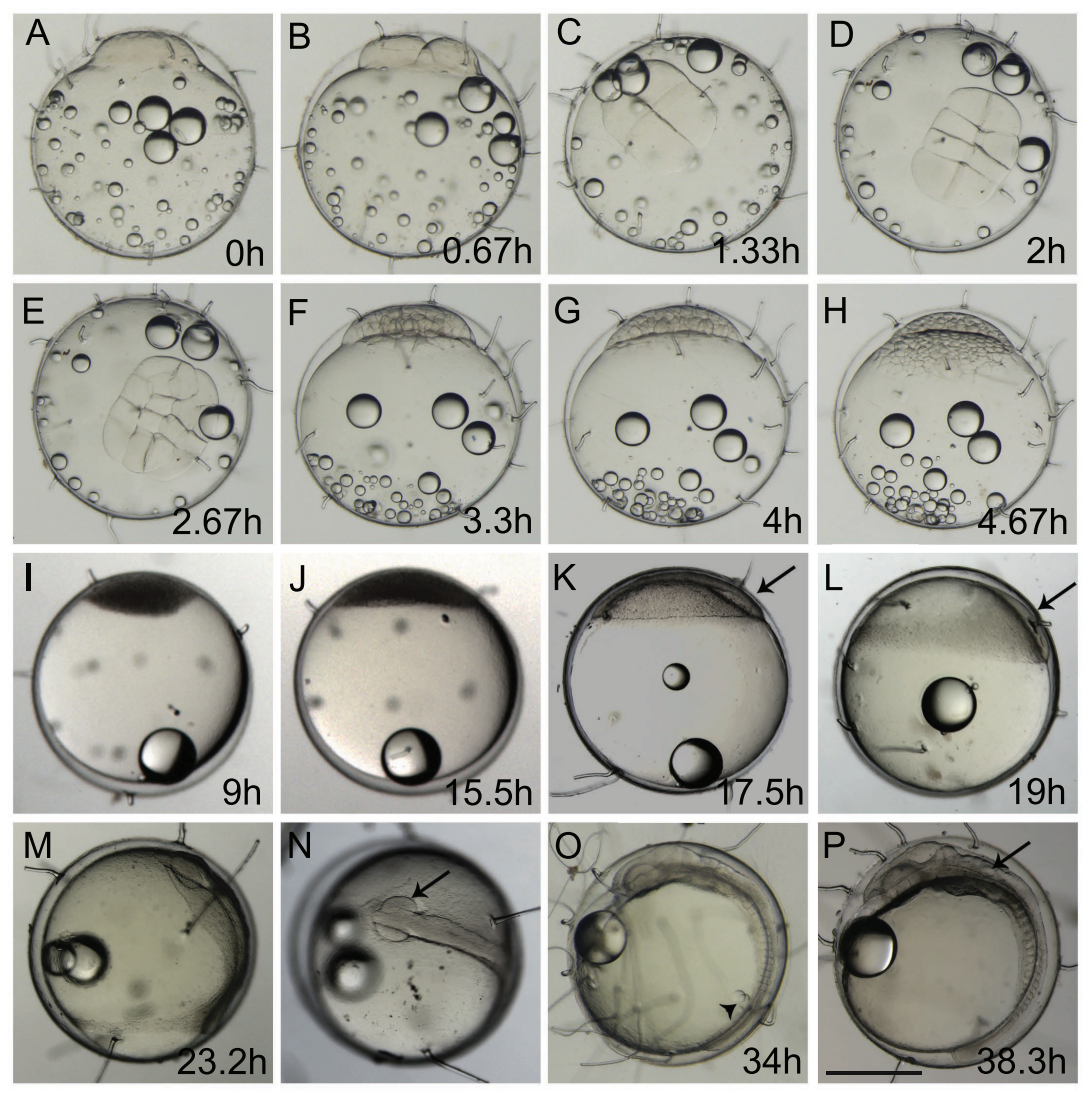
Ontogeny of Brazilian silverside from the 1-cell to 18-somite stage. (A, B, F–M, O, and P) lateral view and (C, D, E, and N) dorsal view. (A) 1-cell stage, considered 0 h from fertilization until first cleavage; (B) 2-cell stage, 0,67 hpf; (C) 4-cell stage, 1.33 h; (D) 8-cell stage, 2 h; (E) 16-cell stage, 2.67 h; (F) 32-cell stage, 3.3 h; (G) 64-cell stage, 4 h; (H) 128-cell stage; 4.67 h; (I) oblong stage; 9 h; (J) sphere stage, 15.5 h; (K) 25% epiboly, 17.5 h the embryo is on the right side (arrow); (L) 40% epiboly, 19 h embryo is on the right side (arrow); (M) 90% epiboly, 23.2 h; (N) optic vesicles forming at 90% epiboly stage (arrow); (O) 14-somite stage embryo, at 34 h showing Kupffer’s vesicle (arrowhead); (P) 18 somite stage, 38.3 h, the otic vesicle is apparent (arrow) (Scale—0.5 mm)

The first cell divisions occurred in rounds of approximately 40 min each at 25 ± 1 °C (Figs. 1A–1H) during the cleavage period. Eggs contained dispersed oil droplets concentrated at the vegetal pole during this period in ±4.6 hpf (Figs. 1A–1H; Video S1). Cells were concentrated at the top of the yolk in the opposite side of the unique oil droplet (Figs. 1I and 1J) during the blastula stage. During the oblong stage at 9.0 hpf (Fig. 1I) and sphere stage at 15.5 hpf (Fig. 1J), the cleavage period generated so many cells that they appeared as a darken mass. At the sphere stage, blastodisc flattening was observed on top of the yolk (Fig. 1J). This stage is the last stage to start the FETm, as it happens before the susceptible gastrulation stage. It was also possible to observe yolk syncytial layer (YSL) cells in the late blastula stage, which were not previously described in *A. brasiliensis* but are present in teleost ontogenesis (Fig. S2). During the late blastula stage, the epiboly process starts and the cells from the blastodisc start to become thinner and spread over the yolk (Figs. 1K and 1L). The percentage of blastoderm coverage of the yolk cell is used to characterize this embryonic stage, as described in zebrafish by Kimmel et al. (1995). The gastrulation stage takes place along the epiboly process and is visible at approximately 16 hpf (Fig. 1K). The blastoderm covers around ¼ of the yolk at 25–30% epiboly and presents a thickening margin where the germ ring appears; there is an accumulation of cells at one position of this ring, known as the embryonic shield (Fig. 1K). At 19 hpf the embryo is at 40% epiboly (Fig. 1L). The embryo at 75–80% epiboly starts to move inside the chorion, showing the reallocation of the oil droplet and the embryonic shield stage, and changing the center of gravity (Video S2). At 23.2 hpf, the embryo at 80–90% epiboly simultaneously starts the neurula stage with the presence of rudimentary optic vesicles and a cell mass anterior to the head (Figs. 1M and 1N).

**Figure 2:**
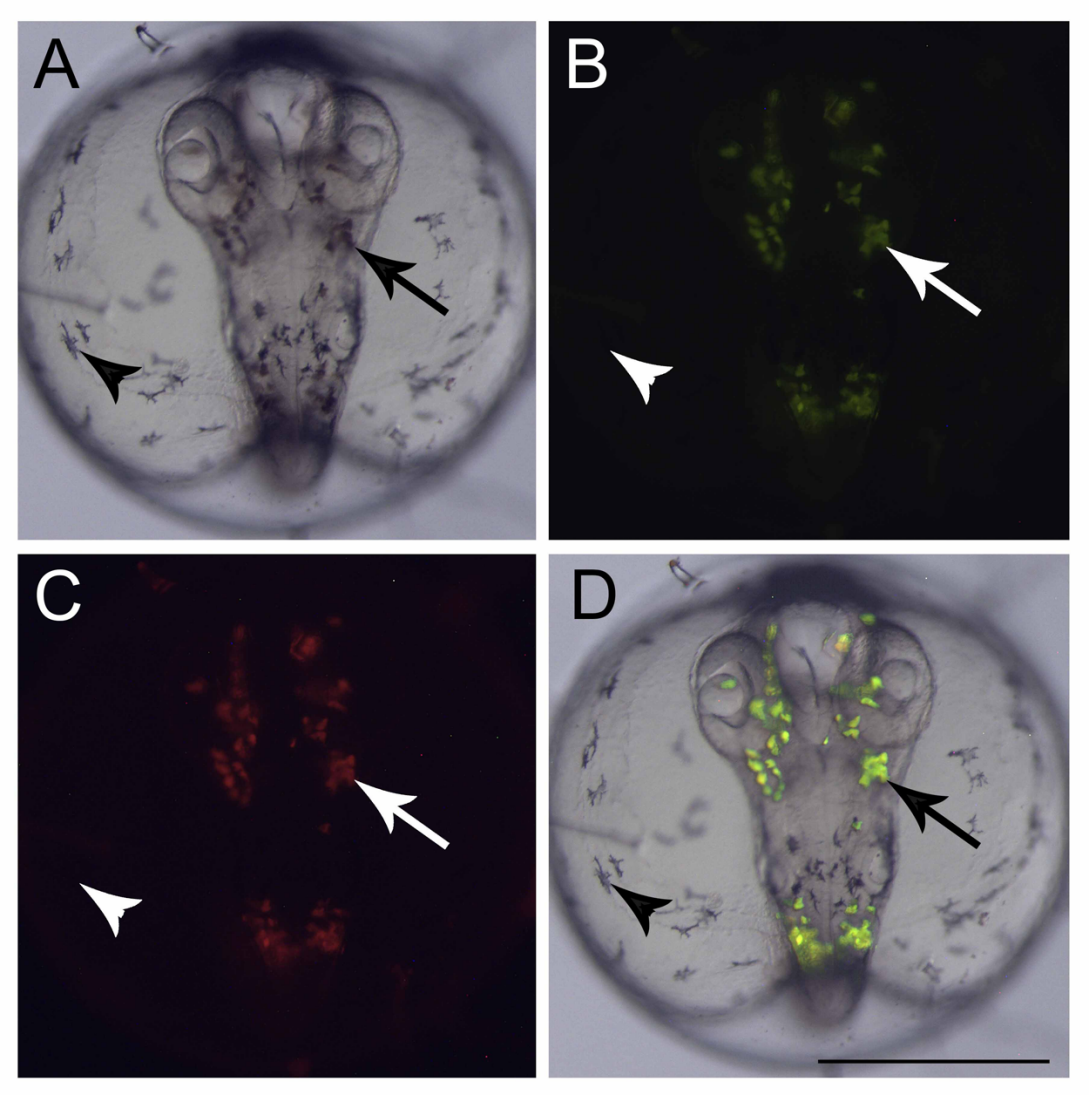
Fluorescent cells, dorsal view. (A) Bright field image showing the pigmented cells: dark brown (arrow) and black (arrowhead), (B) the same embryo image under a GFP filter, (C) dsRed filter and (D) merged images showing that only the dark brown pigmented cells are fluorescent in GFP and dsRED filters. Scale—0.5 mm.

**Figure 3:**
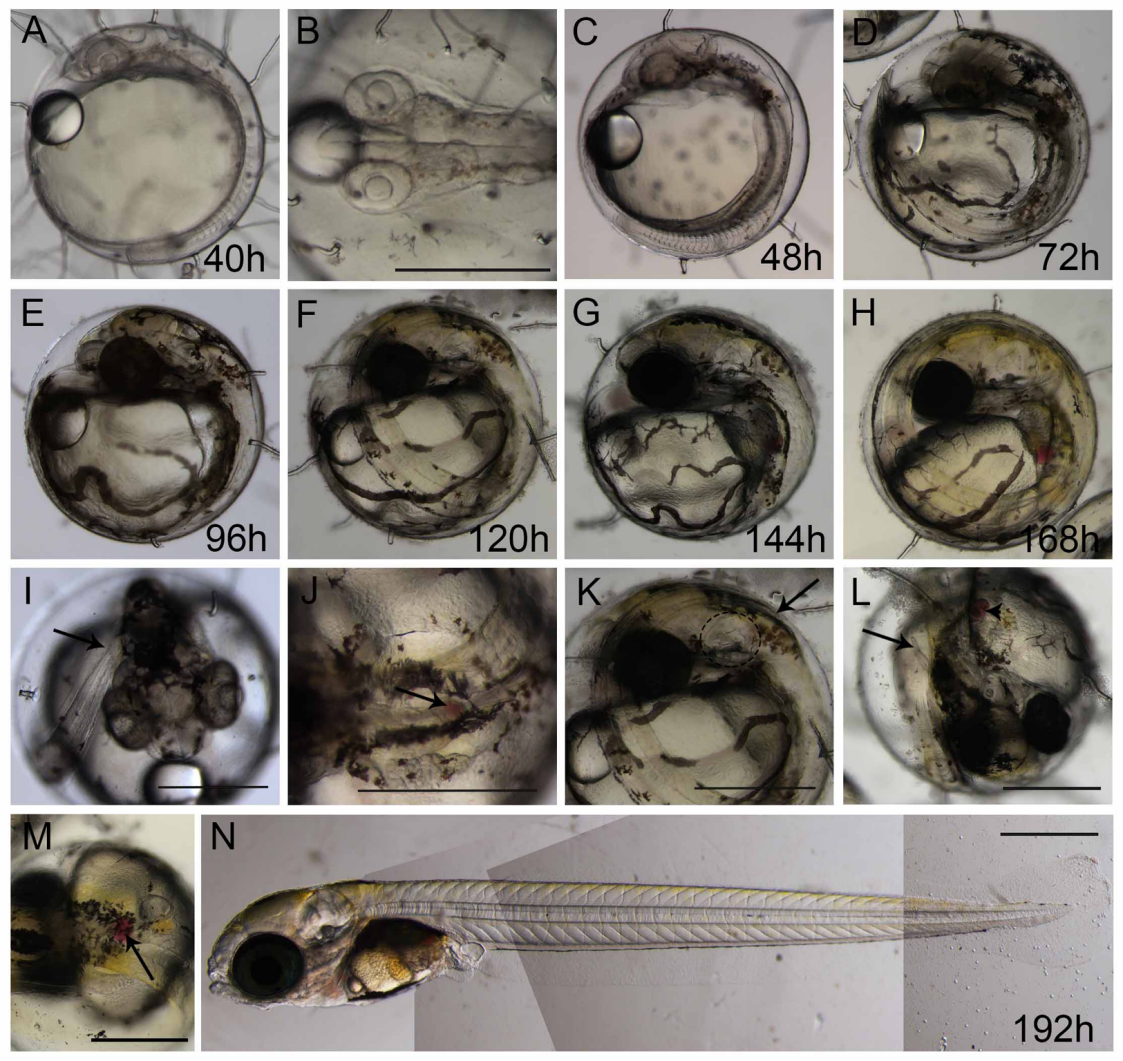
Ontogeny of Brazilian silverside from 20-somite stage until hatching. (A and C–I) lateral view, (B and I) dorsal view. (A) 40 h 20-somite stage; (B) dorsal view of 20-somite embryo with first pigmented cells appearing; (C) 48 h; (D) 72 hpf; (E and I) 96 h in I the arrow shows the tail tip at the level of the otic vesicle; (F, J and K) 120 h or 5 dpf, in J the spleen is visible only in a dorsal view (arrow), and in K, the tail tip (arrow) passes the otic vesicle (dashed ellipse); (G and L) 144 h or 6 dpf; (H and M) 168 h or 7 dpf; (M) dorsal view of 7 dpf larvae showing the spleen as a red spot (arrow); (N) composition picture of a 8-9 dpf hatching larvae. Scale—0.5 mm. The pictures A, C, D, E, F, G, H and N use the same scale bar.

At the end of epiboly, the segmentation stage starts, and somite formation begins. Each pair of somites is formed in rounds of 1 h ± 0.2 h. At 34 hpf, 14 somites can be observed (Fig. 1O) and there are clear morphological changes, such as the development of the Kupffer’s vesicle, and the lenses and brain start to become organized (Fig. 1O). The head is positioned towards the oil droplet from this stage on (Fig. 1O). At 38.3 hpf, during the 17–18 somite stages, the midbrain-hindbrain boundary is starting to be established, and the otic vesicles are present (Fig. 1P). The lenses become visible at the 20 somite stage ±40 hpf. It is possible to observe the first pigmented cells as melanophores, fluorescent chromatophores (Fig. 2), and blood circulation and the heart beat begin at this stage (Figs. 3A and 3B). Pigmented cells of *A. brasiliensis* embryonic/larval development observed from 40 hpf have different types of pigmentation at the transmitted illumination, with black and brownish colors (Fig. 2A). Dark-brown pigmented cells under UV light naturally emit green (Fig. 2B) and red (Fig. 2C) fluorescence. Fluorescent cells overlap with the brown cells but not with the darker black melanocytes (Fig. 2D). This type of autofluorescence of pigment cells in vertebrate embryos has been cited in medaka and is a rare type of cell in nature.

More somites are formed at the posterior end at 48 hpf. Blood circulation is visible, and melanocyte pigmentation is stronger and spread along the anterior-posterior axis and laterally along the surface of the yolk (Fig. 3C); the heart is localized more ventrally and anteriorly to the head. At 72 hpf, the tail elongates and almost reaches the head (Fig. 3D). There is strong pigmentation in the dorsal region of the head, the eyes have darkened, and the melanocytes along the yolk are more organized on top of the blood vessels (Fig. 3D). At 96 hpf, the tip of the tail reaches the otic vesicle (Fig. 3I), the yellow pigmentation, typical of xanthophores, is visible along the dorsal part of the head, and the eyes are strongly pigmented (Fig. 3E). At 120 hpf or 5 dpf, the tail tip passes the otic vesicle (Fig. 3K), the spleen is small and apparent only in a dorsal view (Fig. 3J), and the xanthophores give a yellowish color to the dorsal part of the embryo (Fig. 3F). At 144 hpf or 6 dpf, the internal organs can be observed, as the spleen obtains a red coloration and the tip of the tail reaches the pectoral fins (Figs. 3G and 3L). At 168 hpf, the embryo displays strong pigmentation of the eye, the yellow xanthophores are extensively positioned along the dorsal side of the larvae (Fig. 3H), and the spleen is clearly visible (Fig. 3M). Morphologically, this stage is similar to the hatching larvae. Eclosion at 25 °C occurs between 8 dpf and 9 dpf (Fig. 3N), and the larvae are approximately 4.7 mm. The lipid droplet is still observed in the yolk and this persists for approximately 3 days until it is completely absorbed by the larvae.

### Mortality and hatching rates at different salinities and temperatures

The Brazilian silverside is a euryhaline species. We conducted a static experiment similar to the OECD method for zebrafish FET using one embryo per well in order to observe if salinity could interfere in the embryonic development. The embryos were exposed to salinities ranging from 15 to 35 ppt. One-way ANOVA showed no significant differences in embryo development at different salinities and our results are presented as the mean of the results obtained from embryos exposed to salinities of 15 to 35 ppt at different temperatures.

**Figure 4:**
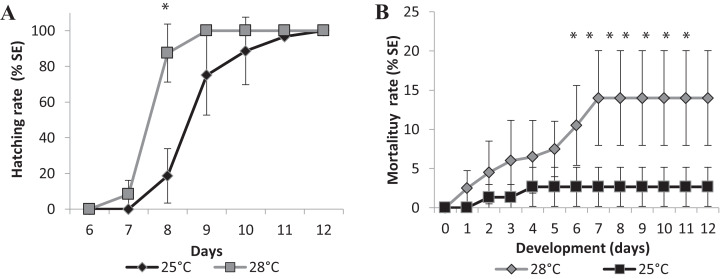
Relative mean of cumulative hatching (A) and mortality (B) rates from all salinities (15-35ppT) tested, in percentage, and ± Standard Errors, at 25 °C and 28 °C. An asterisk (*) indicates significant statistical difference between the temperatures (ANOVA *p* < 0.0001)

Changes in temperature influenced the speed of development as well. We performed two experiments in different temperatures, 25 °C as room temperature and 28 °C, which is the standard temperature for zebrafish development, to investigate if it would affect the eclosion rate. Larvae hatching started earlier for the embryos incubated at 28 °C compared to those at 25 °C. Figure 4 represents the mean eclosion and mortality, at different salinities temperatures. Larvae eclosion started at 7 dpf in the experiments performed at 28 °C. At 8 dpf, 87 ± 16% of the larvae had hatched and 100% of eclosion was observed at 9 dpf (Fig. 4A). In the experiments performed at 25 °C, the embryos took 1 day longer to hatch at 8 dpf, and almost 100% of hatching occurred at 11 dpf (Fig. 4A). There was a significant statistical difference in hatching time and frequency between 25 °C and 28 °C treatments, particularly at 8 dpf (*p* < 0.0001).

We observed a significant difference in mortality in the embryos raised in different temperatures. The accumulated mortality of the embryos at 25 °C was 2.67 ± 2.49% (Fig. 4B). However, at 28 °C, the mortality was significantly higher than at 25 °C (ANOVA *p* < 0.0001) starting on the sixth day postfertilization and reaching 14.00 ± 26.04% at the end of experiment (Fig. 4B). Higher temperatures may interfere with the survival rate of embryos, and FETm should not be carried out at 28 °C.

Embryos started to die approximately 4 days after hatching, on day 13 (data not shown), indicating that embryo mortality must be scored until day 12. We established 25 °C as the standard temperature for the Brazilian silverside FETm, and determined that testing should be completed in 12 days.

### Toxicity tests

**Figure 5:**
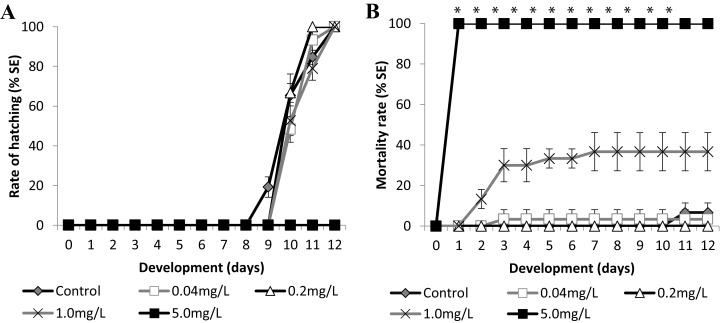
Brazilian silversides embryos exposed to different SDS concentrations. **** (A) Hatching rate, in percentage, in mg·L^−1^. (B) Mortality rate (±Standard Error). (A and B) The graphs correspond to cumulative data. An asterisk (*) indicates significant difference between the values on the corresponding day (ANOVA *p* < 0.0001).

**Figure 6:**
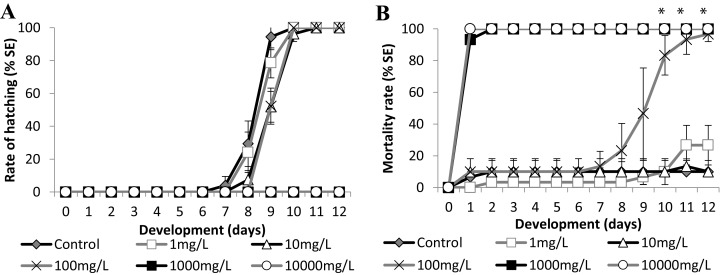
Brazilian silversides embryos exposed to different Zn concentrations. (A) Hatching rate of live embryos, in percentage, in mg·L^−1^. (B) Mortality rate of embryos, in percentage (± Standard Error), in mg·L^−1^. (A and B) the graphs correspond to cumulative data. An asterisk (*) indicates significant difference between the values on the corresponding day (ANOVA *p* < 0.001).

We used two known reference substances, the surfactant sodium dodecyl sulfate (SDS), and the metal zinc, as zinc sulfate heptahydrate, at salinity 35 ppt to validate the experiments and to analyze the Brazilian silverside embryo sensitivity. Control embryos hatched showing wild-type morphologies at 9 dpf and mortality lower than 10% until the end of the test in both experiments, validating the tests such as it is considered for FET tests (Braunbeck et al., 2005; Setiamarga et al., 2008) (Figs. 5 and 6). The FET OECD test guideline features coagulation, lack of somite formation, non-detachment of the tail, and lack of heartbeat as lethal endpoints (OECD, 2013). Only coagulation was observed along the tests and was used to calculate LC50.

### SDS

*Atherinella brasiliensis* embryos were exposed to different concentrations of SDS, from 0.04 to 5.00 mg/L^−1^, over 12 days. At 24 h, the LC50 was 1.31 mg/L^−1^, was 1.08 mg/L^−1^ (±0.14) at 96 h and 1.01 mg/L^−1^ (±0.19) at 12 days. The hatching rates among the different concentration treatments were similar to the control throughout the experiment, with the exception of 5.00 mg/L^−1^ SDS (Fig. 5A).

Embryos exposed to the lower concentrations 0.04 and 0.2 resulted in no significant difference in mortality (Fig. 5B). Embryos died in the 1.00 mg/L^−1^ SDS treatment, showing 36.67 ± 9.43% mortality at the end of the experiment (Fig. 5B). This mortality was significantly different from that of the control (*p* < 0.0001). The treatment with 5.00 mg/L^−1^ of SDS led to 100% lethality at 24 hpf (Fig. 5B) showing a sensitivity of the embryos to SDS on the first day of development.

### Zinc

Embryo exposure to the lowest Zn concentrations (1.0 and 10.0 mg/L^−1^) showed no difference in the hatching rate nor in the mortality rate compared with those of the control (Fig. 6). Although the embryos exposed to 100.0 mg/L^−1^ showed no differences in the eclosion rate compared to the control (Fig. 6A), this treatment showed increasing mortality close to the stage of hatching, leading to over 90% dead larvae by 11 dpf (Fig. 6B) (ANOVA *p* < 0.001). The hatched embryos died 1 day after leaving the chorion at this concentration. Finally, embryos exposed to 1,000 mg/L^−1^ and 10,000 mg/L^−1^ died in the first 24 h of treatment (Fig. 6B). Importantly, the exposure of the embryos to Zn showed that they were sensitive to this trace metal at two different developmental stages. During the first days of development the observed LC_50_ were 221.64 (±90.32) mg/L^−1^ at 24 h and 153.94 (±18.59) mg/L^−1^ at 96 h and after the hatching period at 12 days was 12.64 (±3.95) mg/L^−1^ (Fig. 6B).

## Discussion

This study presents the Brazilian silverside *A. brasiliensis* as a prominent test species for a fish embryo test (FET) in marine conditions. We named the test the marine FET or FETm. The zebrafish is considered a great model species, but FETs cannot be performed in different salinities using this fish species. Thus, there is a need for methods to evaluate lethal and sublethal toxicities of substances in the environment and their effects on organisms in a brackish or marine environment. In South America and Brazil, there is no native fish species for this type of test. The Brazilian silverside displays many interesting characteristics which make it valuable as an alternative model organism for studying ecotoxicology.

### Adult maintenance and sexual maturity

The diet of the fish in the laboratory with commercial flocculated and granulated food was well accepted considering the early larval stages and the opportunistic feeding behaviors of adults (Chaves & Vendel, 2008; Contente, Stefanoni & Spach, 2010; Rocha, Silva-Falcão & Severi, 2008). We obtained embryos from individuals captured in their natural environment after 2 to 3 weeks of acclimation in an aquarium. The first maturation occurred according to the environment (Favaro, Lopes & Spach, 2003) at 8.0–9.0 cm in length (Bervian & Fontoura, 2007). However, this size is obtained at 1 year of age in the natural environment, while in the laboratory, it took close to 9 months. Development was faster in the laboratory possibly because temperature and water conditions were kept stable year-round and were coupled with regular food supply. In the natural environment of the Paranagua estuarine area in southern Brazil and the Imboassica Lagoon in the southeastern part of the country, populations are found in a proportion close to 1:1 female:male during autumn, summer, and winter but 2:1, respectively, in spring (Cardoso, 2007; Fávaro, Oliveira & Verani, 2007). A range of 10–15 fish was maintained in the aquarium of 130 L, which can be maintained in most research laboratories. The released eggs settled on artificial plants and in the filter drains, which can be improved with the development of different material surfaces where the eggs could attach, thus avoiding adult cannibalism and the possibility of contamination.

### Natural egg laying and embryo preparation

The first report of Brazilian silverside embryos was in 2005. Del Río et al. (2005) obtained gametes by performing abdominal massage on the adult fish, and the eggs were fertilized by manipulation. In another report, Brazilian silverside larvae were obtained from ichthyoplankton samples (França et al., 2007). We developed several technical improvements: first, no hormones were used to induce egg laying, and no massage was used to obtain the gametes; second, the eggs were fertilized naturally and were collected from the aquarium two to three times per week; third, because we did not manipulate and stress the adult fish, embryo viability was increased. Del Río et al. (2005) found that the percentage of larvae hatching was only 36.03 ± 8.96%. This low percentage of larval hatching could have been due to manipulation during egg separation and/or cleaning of the eggs before the test. Microorganismal growth around the chorion may have led to a decrease in the oxygen rate consequently affecting embryo development and hatching. The filaments along the chorion are long and sticky making it a suitable microenvironment for microorganism growth. We removed the long filaments before the beginning of the tests by cutting the filaments with the scissors after they were pulled taut with tweezers. Embryos were easily generated for the tests. In Del Río et al. (2005), the embryos were raised in higher temperatures of 26 ± 2 °C, and hatching occurred 2 days earlier on average (day 6), than in the present study at 24 ± 1 °C. However, higher temperatures may lead to an increase in mortality as shown in our results and discussed later in the topic: Experimental conditions. Altogether demonstrate that our technical improvements make the Brazilian silverside embryos easy to be obtained, and proper cleaning prior to the experiments is essential for experimental success.

### Embryo development and fluorescent cells

Morphological analysis of the Brazilian silverside embryos conducted after embryo preparation revealed a transparent embryo covered by a transparent chorion. Brazilian silverside embryos have a similar morphology to medaka embryos, making it easier to identify their internal structures (Iwamatsu, 2004). Silverside embryos show lipid droplets, similar to medaka, but they develop at least a day faster. The embryos and larvae develop and hatch in 8 to 9 days at 25 °C while medaka hatch in 10 days (Iwamatsu, 2004). The neurogenesis of the Brazilian silverside happens before the end of the epiboly stage, but the posterior structures, including the tail, develop after the anterior region and not simultaneously when compared to zebrafish embryos (Kimmel et al., 1995). The blastoderm covers approximately ¼ of the yolk at a 25–30% epiboly and possess a thickening margin. In this margin, the germ ring appears and the accumulation of cells is called the embryonic shield (Fig. 1K). The development of the germ ring occurs earlier than in the zebrafish and is similar to medaka embryonic development. The embryonic shield is preparing for gastrulation and is observed during 50% epiboly or the shield stage (Iwamatsu, 2004; Kimmel et al., 1995). At 23.2 hpf, the embryo at 80–90% epiboly simultaneously starts the neurula stage with the presence of rudimentary optic vesicles and a cell mass anterior at the head (Figs. 1M and 1N). Eye formation is similar to that in medaka embryos, while in zebrafish embryos, the optic primordium appears later, at the 8-somite stage, when the epiboly movements have already ceased (Iwamatsu, 2004; Kimmel et al., 1995).

An interesting observation from the Brazilian silverside embryos was the fluorescent cells observed during embryogenesis. To date, the species studied in the superorder Atherinomorphae such as the silversides *M. menidia*, *M. beryllina*, and Indian medaka and *O. melastigma* do not have records of fluorescent chromatophores. However, in *O. latipes* pigmentation, specific cells show autofluorescence under the UV-light (Iwai et al., 2009; Loire et al., 2013). Those cells are named leucophores and in some teleost fish species the adults present a white color and the embryos an orange color (Fujii, 1993; Lewis et al., 2019; Oliphant & Hudon, 1993). The freshwater medaka and Brazilian silverside embryos presented in this work have been shown to auto-fluoresce under UV-light. *A. brasiliensis* embryo fluorescent cells could originate from neural crest cells, as do the medaka’s leucophores, indicating that they may be closely related to xantophore differentiation. This differentiation depends on the fine gene regulation of *sox5* and *pax7a* genes (Kimura et al., 2014; Nagao et al., 2014), which could be determined in future studies. Setiamarga et al. (2008) showed that medakas are more highly derived than silversides (Setiamarga et al., 2008). Among the Atherinopsidae family, Brazilian silversides display a more ancestral position in the phylogenetic tree than *M. menidia* (Bloom et al., 2013). Biofluorescence has been discussed in evolution more recently in the cladus subseries, Ovalentariae. Red and green fluorescent cells have been observed in the superorder Bleniimorphae in adult fish. However, in superorder Atherinomorphae, only adults of the family Melanotaeniidae, a representative of the order Atheriniformes, and the family Poeciliidae, show green fluorescence (Sparks et al., 2014). It is possible that the generation of red and green fluorescent cells may be an ancestral characteristic of Atherinomorphae which was retained in the Brazilian silverside. It is possible that not all phylogenetically important species with biofluorescence have been identified, which hinders the understanding of the evolution of fluorescent cells in fish. Ours is the first study on these fluorescent cell types in atherinid fish embryos. These fluorescent cells may be used as natural markers for neural teratogenicity, or in studies of neural crest derived cell migration. For example, a substance that affect the neurogenesis or neural-crest cell differentiation may present impaired leucophore differentiation or migration. The lack of fluorescence or impaired leucophore migration may be an interesting endpoint to a specific toxic effect aside from any cranio-facial or curved-tail endpoint. In *A. brasiliensis*, there is no need for transgenic lines to observe those cells.

### Experimental conditions: salinity, temperature, and time duration

Brazilian silverside embryos may be used to establish an ecotoxicological method test in South America using a local species from neotropical regions. Salinity and temperature are important variables for some compounds. Here, the marine fish embryo test (FETm) was standardized to be similar to the FET and it was observed that changes in salinity, from 15–35 ppt, did not affect embryonic development. However, temperatures of 28 °C may negatively influence survival and embryonic hatching time. Temperatures of 28 °C decreased the hatching time from 8 to 9 days to 7 to 8 days. However, an increase in mortality to approximately 20% of the embryos demonstrated that this test should not be conducted at this temperature. The increased mortality of Brazilian silverside embryos incubated at 28 °C indicates that this temperature might be close to the thermal tolerance of the organism. Testing should cease on day 12 because approximately 4 days after hatching, on day 13, embryos began to die likely as a consequence of absorbing the yolk and demanding oral food intake.

Brazilian silverside eggs hatch at 8–9 days postfertilization in temperatures of 25 °C. This hatching time is the same or faster when compared to hatching times of other marine or euryhaline species. For example, the Indian medaka takes 20 days to hatch at 26 °C in 90 mm petri dishes, using 20 mL of artificial sea water (Wu et al., 2012). Standard test methods developed by the American EPA use larvae of the Atlantic silverside *M. menidia* and Inland silverside *M. beryllina* instead of embryos. Embryos of the sheepshead minnow *C. variegattus* hatch in 6–7 days at temperatures of 25 °C (USEPA, 2002a, 2002b, 2002c). Atlantic haddock embryos incubated in a 50 L beaker at 7–8 °C begin hatching at 13 dpf, and 50% of the embryos hatched at 14 dpf (Sørhus et al., 2015, 2017). Embryos of the Pacific herring *C. pallasii* can be obtained artificially and the hatching time varies between 14 dpf and 20 dpf in a 90-L aquarium maintained at 9.1 ± 0.1 °C (Incardona et al., 2015). They may also hatch on day ten at 12 °C (Dinnel et al., 2011). Brazilian silverside embryos are easy to obtain, lay eggs throughout the year with stable parameters as described in the methodology, and the duration of embryonic testing is reliable and similar or faster than that of other marine or euryhaline species previously described.

### Embryo sensitivity to reference substances

We used two known reference substances, sodium dodecyl sulfate (SDS) and zinc, to determine the sensitivity of the Brazilian silverside. SDS is a surfactant used as a reference toxicant for acute toxicity testing using effluents in North America and for ecotoxicological studies with fish and other animal species in Europe and Brazil (Gibson et al., 2016; Jorge & Moreira, 2005; Tornambè et al., 2018; USEPA, 2002c). We conducted testing at a salinity of 35 ppt to understand the toxicity of SDS in marine samples. *A. brasiliensis* is an euryhaline species and further experiments are needed to evaluate the toxicity of SDS to the Brazilian silverside at different salinities. The tests at a salinity 35 ppt demonstrated that *A. brasiliensis* embryos were sensitive to SDS at 96 h with an LC_50_ of 1.08 (± 0.14) mg/L^−1^. The Brazilian silverside is as sensitive as *D. rerio* embryos at an LC_50_ of 3.46 mg/L^−1^ (Aalders et al., 2016), *P. promelas* at an LC_50_ of 8.6 mg/L^−1^ (USEPA, 2002c), and *M. beryllina* larvae at an LC_50_ of 4.81–5.34 mg/L^−1^ (USEPA, 2002b). *A. brasiliensis* embryos were more sensitive than the euryhaline crustacean *Tigriopus. fulvus* at an EC_50_ of 7.42 mg/L^−1^ ± 0.73, and as sensitive as the embryos of the sea urchin species *Arbacia. lixula*, *Paracentrotus. lividus*, and *Sphaerechinus. granularis* at 28–38 h, CI_50_ 1.55 mg/L^−1^ ± 0.17; 1.48 mg/L^−1^ ± 0.34 and 1.59 mg/L^−1^ ± 0.22 respectively (Cesar et al., 2004; Mariani et al., 2006). Zinc is an essential trace metal that can be toxic in high concentrations. A review from 1995 analyzed data regarding the influence of salinity on the toxicity of various classes of chemicals and indicated that euryhaline species are more resistant to substances at isosmotic salinities and were more sensitive to extremes in salinity (Hall & Anderson, 1995). Later stages of development are more sensitive than the initial stages, as was seen in zebrafish in 1965 (Skidmore, 1965). The LC_50_ of the Brazilian silverside embryos exposed to Zn was 221.64 (±90.3) mg/L^−1^ at 24 h and 153.94 (±18.59) mg/L^−1^ at 96 h. However, larvae died when exposed to Zn 100 mg/L^−1^ during or a few hours after hatching. The LC_50_ dramatically decreased to 12.64 (±3.95) mg/L^−1^ on day 12. Larvae from two euryhaline species (7–8 day-old *F. heteroclitus* larvae with an LC_50_ at 96 h of 34.5 mg/L^−1^ and *K. marmoratus* with an LC_50_ at 96 h of 27.88 mg/L^−1^) showed similar LC_50_ values after 96 h exposure when exposed to Zn in 36 ppt saltwater (Bielmyer et al., 2012). According to an EPA report from 1987, the acute toxicity test for saltwater fish from nine species, including the Atlantic silverside, ranged from 2.7–83 mg/L^−1^ (USEPA, 1987). The chorion may protect the embryo against some substances, which has occurred in zebrafish embryo testing in Braunbeck et al. (2014).

It would be interesting to establish sublethal endpoints for the Brazilian silverside for FETm in addition to the lethal endpoints. However, our reference tests did not determine sublethal points and future experiments with positive control for FET such as 3,4-dichloroaniline should be conducted (Busquet et al., 2014). Nevertheless, we adapted a table for lethal and sublethal endpoints based on the morphological characteristics of *A. brasiliensis* embryo and larvae (Table S1). This table was based on the endpoints suggested by Lammer et al. (2009) and used large morphological structures of the embryos as parameters for a detailed analysis. For example, somite morphology was used as a toxicological endpoint, while the spleen was not. Our table makes the FETm a practical approach for different ecotoxicological testing analyses based on the results of our SDS and Zn embryo tests.

## Conclusions

The Brazilian silverside is the first South American species established for fish embryo ecotoxicological testing. It meets technical criteria such as easy maintenance in the laboratory and reproductive potential. Eggs are laid naturally in the aquarium and can be easily collected and cleaned for testing. Embryos are transparent, can be promptly analyzed, and hatch at similar times as other species used for ecotoxicological testing. Furthermore, embryos displayed natural fluorescent cells and were sensitive to reference substances. Overall, the Brazilian silverside *A. brasiliensis* can be considered a relevant test species for a marine fish embryo test.

## Supplemental Information

10.7717/peerj.11214/supp-1 Supplemental Information 1 Raw data of the experiments. Mortality and hatching of embryos exposed to different temperatures and salinities, and embryos exposed to SDS and zinc.

10.7717/peerj.11214/supp-2 Supplemental Information 2 Video 1: Eggs contained dispersed oil droplets particularly concentrated at the vegetal pole during cleavage period.

10.7717/peerj.11214/supp-3 Supplemental Information 3 Video 2: The embryo at 75%-80% epiboly starts to move inside the chorion, showing the reallocation of the oil droplet and the embryo, changing the gravity center.

10.7717/peerj.11214/supp-4 Supplemental Information 4 Supplemental Figure and Table.

## Additional Information and Declarations

### Field Study Permissions

The following information was supplied relating to field study approvals (i.e., approving body and any reference numbers): Permissions for fish collection were obtained from the Brazilian Ministry of the Environment (MMA)/Chico Mendes Institute for Biodiversity Conservation (ICMBio) (Permits n° 43874-1 and 43874-2).

### Data Availability

The following information was supplied regarding data availability: The raw measurements are available in the Supplemental File.
