# Supplementary material for: Brazilian silverside, Atherinella brasiliensis (Quoy & Gaimard,1825) embryos as a test-species for marine fish ecotoxicological tests
Source: PeerJ. 2021 Apr 14;9:e11214. doi: 10.7717/peerj.11214 (PMC8052962; doi:10.7717/peerj.11214)
Supplement: Supplemental Information 4 [file peerj-09-11214-s004.pdf]

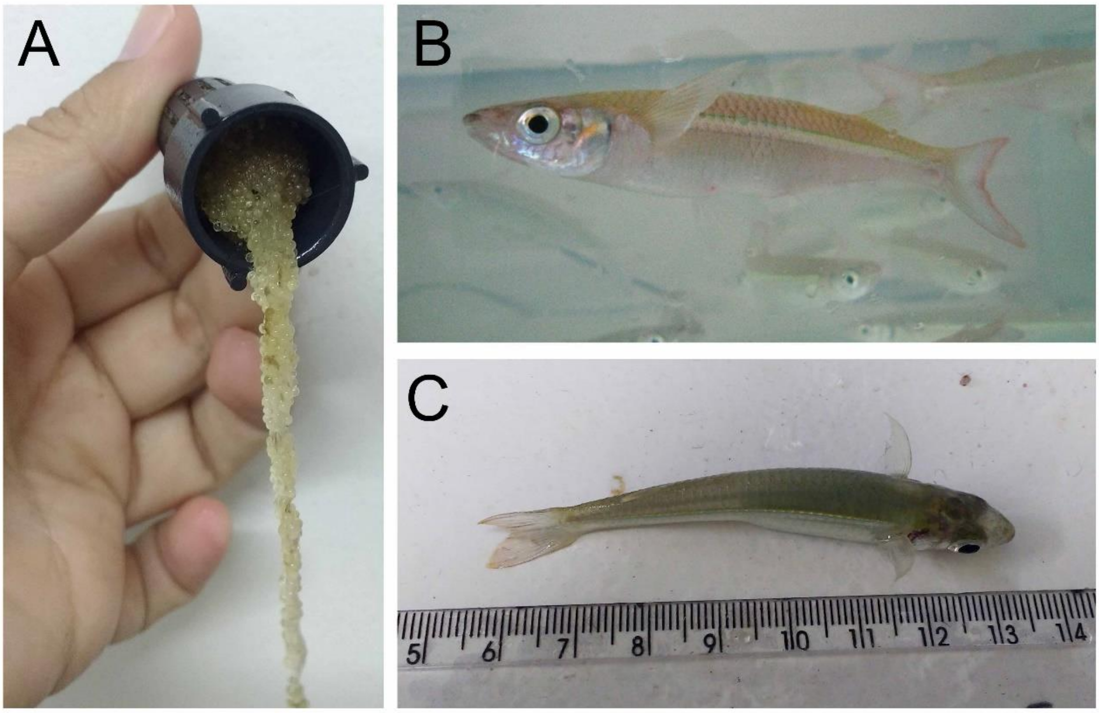

Supplemental figure 1- (A) Egg cluster in the filtration net; (B) adult specimen of *A. brasiliensis,* Brazilian silversides, in the aquarium; (C) mature Brazilian silverside measurement.

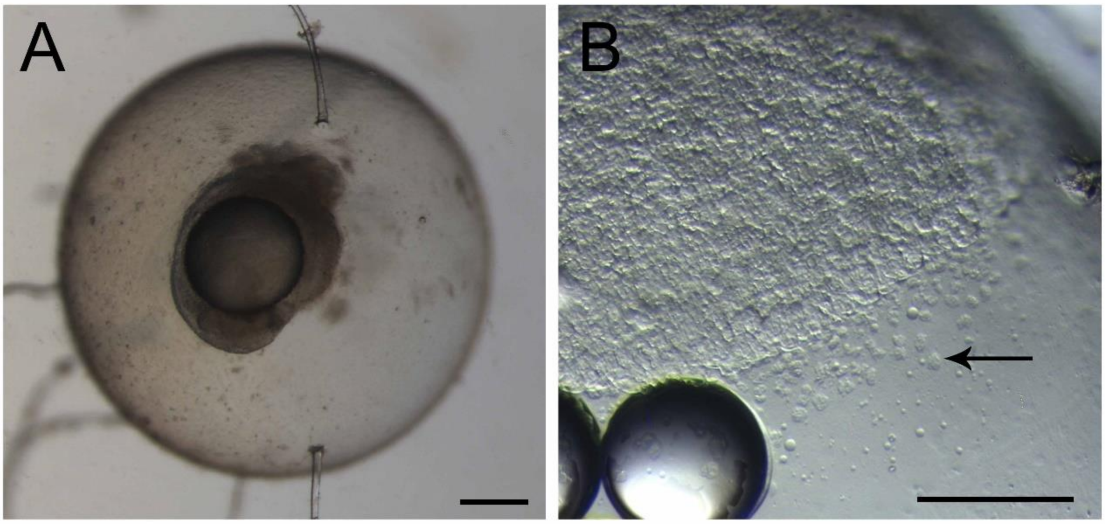

Supplemental figure 2- (A) Dead embryo coagulated under brightfield illumination; (B) yolk syncytial layer during epiboly and arrow showing the large cells at the edge of epiboly. Scale 0,2 mm.

Toxicological endpoints

Regarding Brazilian silversides morphology during development, toxicological endpoints are suggested below in order to perform MFETs (Table S1). The endpoints are similar to those suggested by Lammer *et al.* 2009. The table can be used in any technical approach and is subdivided into three main groups: lethal endpoints, sublethal developmental endpoints and teratogenicity endpoints.

Lethal endpoints: morphological characteristics of the embryos that implies embryo death.

Coagulation: Embryos die and tend to form a concentrated amorphic mass that is opaque under translucent light in the middle of the chorion (Sup. Fig 2). This coagulation feature is more common in the first days, but this observation of embryo death and degeneration can be observed until the last day the embryo/larval day of the test.

Tail deformation: This includes any kind of tail deformation, torsion or impairment of tail growth will make it stay close to the chorion and could be a result of somite or notochord malformation. In the case of zebrafish, this endpoint was tail detachment, regarding the elongation of the yolk underneath the tail, but in the Brazilian silversides, this elongation of yolk does not exist; thus, we characterize it as tail deformation that can be analyzed until day 12.

Absence of somite formation: This occurs when it is not possible to observe somite development from 27 hpf until 48 hpf. The somites are well defined, and any malformation is easily observed on those stages and later.

Absence of heart beat: This is considered when after 5 seconds of observation, the heart does not contract.

Absence of hatching: This is considered as occurring after 11 days.

Sublethal developmental endpoints

Completion of gastrula: It should be observed until the end of epiboly movements. It is normally observed in 24 hpf at 25°C.

Formation of somites: This is the deformed shape of or undefined separation of the somites from 27 hpf until 48 hpf. The somites are well defined, and any malformation is easily observed during those stages. This endpoint is considered teratogenicity.

Development of the eyes: The retina and lenses are easily observed if the retina has a round-shape and the position of the lenses symmetrically in the middle of the retina.

Heartbeat / blood circulation: The frequency of the heart beat can be considered a great morphological endpoint. Changes in the frequency and presence of clots in the tail, body or brain can be easily analyzed but should not be confused with the spleen that also appears after 9 days as a round red organ. Any changes in the pericardium can also indicate a heart/blood circulation endpoint.

Pigmentation: The pigmentation of xanthophores (the yellow color) but especially the distribution of melanophores can be used as endpoints.

Erratic swimming or no reaction to touch: Swimming may indicate disruption of neurological features and the balance of the larvae.

-Teratogenicity endpoints:

Teratogenic Endpoints: These are malformations of visible structures of the embryos as otoliths (number of otoliths in the otic vesicle), craniofacial (as small or longer mandible), heart (epicardial chamber same as heart edema, thin atrium and ventricle) and tail (curved tail).

Table S1. Endpoints considered in the embryo testing with Brazilian silversides.

**Endpoints**	**Exposure time**					
Lethal endpoints	8 h	24 h	48 h	72 h	4-9 days	10-12 days

Coagulation	*	*	*	*	*	*
Tail deformation				*	*	*
No somite formation		*[1]	*	*	*	*
No heart beat			*	*	*	*
No hatching						*
Sublethal developmental endpoints	8 h	24 h	48 h	72 h	4-9 days	10-12 days
Completion of gastrula	*	*				
Formation of somites		*	*	*		
Development of the eyes		*	*	*	*	
Heartbeat/blood circulation				*	*	
Pigmentation			*	*	*	
Erratic swimming or no reaction to touch						*
Teratogenicity endpoints	8 h	24 h	48 h	72 h	4-9 days	10-12 days
Craniofacial malformation				*	*	
Malformation of otoliths			*	*	*	
Malformation of tail				*	*	
Malformation of heart (edema formation and beat)				*	*	
Yolk deformation					*	*
General growth retardation		*	*	*	*	

*Endpoints modified from Lammer *et al.* 2009 1- somite formation from 27 hpf.
